# Preliminary Evidence for Autoimmune Regulator Occupancy at Promoter Regions of Known Autoantigens in Human Peripheral Lymphocytes Obtained by Chromatin Immunoprecipitation Assay

**DOI:** 10.3390/ijms27135807

**Published:** 2026-06-26

**Authors:** Caterina Nardella, Irene Mezzani, Eleonora Pace, Alessandra Fierabracci

**Affiliations:** 1 Bambino Gesù Children’s Hospital, Istituto di Ricovero e Cura a Carattere Scientifico (IRCCS), 00146 Rome, Italy; caterina.nardella@opbg.net (C.N.); irene.mezzani@opbg.net (I.M.); eleonora.pace@opbg.net (E.P.); 2 Life Sciences, Health and Health Professions Department, Link Campus University, 00165 Rome, Italy

**Keywords:** autoimmune regulator (AIRE), central tolerance, peripheral tolerance, autoantigens, gene promoters, extrathymic AIRE-expressing medullary thymic epithelial cells, tolerogenic dendritic cells

## Abstract

Central tolerance is provided by the autoimmune regulator (AIRE) expressing medullary thymic epithelial cells through high-avidity recognition of self-antigens. Peripheral mechanisms regulate adaptive immunity by deleting autoreactive T-cells that escape thymic selection, or by inducing their functional unresponsiveness through interaction with antigen-presenting cells, exposing cognate antigens. Multiple types of extrathymic AIRE-expressing cells residing in secondary lymphoid organs have been consistently described. We investigated whether AIRE binds to promoters of known autoantigens in human peripheral blood mononuclear cells by chromatin immunoprecipitation from four healthy donors using an anti-AIRE monoclonal antibody. Quantitative real-time PCR was used to detect AIRE occupancy at promoters of selected autoantigens. Accordingly, we revealed promoter amplicons of all tested autoantigen genes, and their corresponding transcripts. Expression of AIRE was further confirmed at both transcriptional and protein levels. Overall, although the role of AIRE in regulating autoantigen expression in thymic epithelial cells has been well reported, our study provides preliminary descriptive evidence of AIRE occupancy at promoters of known autoantigens in human bulk peripheral blood mononuclear cells, suggesting the presence of AIRE-mediated regulatory events in peripheral blood. Our data provide a rationale for future investigations aimed at elucidating the underlying molecular and immunological mechanisms.

## 1. Introduction

The etiopathogenesis of human autoimmunity is not fully elucidated. Several mechanisms underlying the occurrence of adaptive and innate immune responses are still under investigation [1]. The adaptive immune system consists largely of T-cells, which recognize specific epitopes derived from antigens through their T-cell receptors (TCRs). The recombination of key TCR DNA coding segments allows for an essentially endless repertoire of potential antigen-binding TCRs [2,3].

The autoimmune regulator (*AIRE*) gene encodes for a non-classical transcriptional factor, namely, a facilitator/modulator that is expressed in medullary thymic epithelial cells (mTECs), where it plays a pivotal role in the process of negative selection occurring in the perinatal age [4]. With regard to mature thymocytes, these are screened for autoreactivity and, if recognition of high avidity in self-antigens occurs, they are clonally eliminated [5]. Nevertheless, some self-reactive T-cells can escape from the thymus, enter the peripheral blood and become activated during the patient’s lifetime, generating autoimmune diseases under specific circumstances of genetic/environmental interaction. Indeed, in the human monogenic disorder Autoimmune PolyEndocrinopathy-Candidiasis-Ectodermal Dystrophy syndrome (APECED, Autoimmune Polyendocrine Syndrome Type 1 (APS1)) caused by AIRE deficiency or loss-of-function *AIRE* mutations, T-cell autoreactivity is responsible for dysfunction of several endocrine glands, as well as non-endocrine issues. A clinical diagnosis is made where there are at least two out of the following three relevant manifestations: hypoparathyroidism, adrenocortical insufficiency and chronic mucocutaneous candidiasis [4].

It is generally recognized that the mechanism of central tolerance provided by the AIRE-expressing mTECs is not the sole factor in the regulation of the adaptive immune response, because peripheral tolerance mechanisms exist. In the peripheral lymphoid organs, the deletion or induction of functional unresponsiveness in autoreactive T-cells requires interaction with an antigen-presenting cell (APC) expressing cognate antigens. Peripheral tolerogenic dendritic cells (DCs) can induce anergy, or peripheral clonal deletion of T-cells, by presenting an antigen without expressing the costimulatory molecules CD80 and CD86 necessary for the full activation of T lymphocytes [6]. Furthermore, cell-extrinsic peripheral tolerance mechanisms involve suppression of effector cells by regulatory phenotypes [7].

In addition to AIRE expression in thymic medullary epithelial cells, multiple types of extrathymic AIRE-expressing cells (eTACs) [8] have been reported, residing particularly in secondary lymphoid organs. This suggests a putative immune regulatory function, a suggestion which was confirmed by the expression of immunomodulatory molecules such as IDO (indoleamine 2,3 dioxygenase), IL-10 (interleukin 10) and PD-L1 (programmed death-ligand 1) [9]. In particular, eTACs possess antigen-presenting features, as shown by markers that largely overlap with DCs, possibly representing a distinct subgroup [8,10]. They have a potential function in peripheral tolerance through antigen presentation via MHC (major histocompatibility complex) class II-TCR. Suzuki et al. detected low AIRE expression in B-cells (CD19^+^), cells from the monocyte/macrophage lineage (CD14^+^) [11] and stromal cells [12], whereas T-cells (CD65^−^ CD14^−^ CD19^−^) tested negative [11]. eTACs have indeed been detected in inflamed tissues of patients with autoimmune diseases and in various cancer tissues [8].

Recent findings indicate that Aire controls immune tolerance through an additional mechanism: the induction in the thymus of FoxP3-positive regulatory T-cells (Tregs) that have the ability to suppress autoreactive cells. Thus, Aire deficiency also limits the development of Tregs and/or makes them dysfunctional [13].

AIRE is a transcription factor with many targets, leading to different responses in different contexts. Therefore, the exact role of AIRE expression in peripheral tolerance still needs to be unraveled. In the light of the foregoing, we set up experimental conditions using chromatin immunoprecipitation (ChIP) of human peripheral blood mononuclear cells (PBMCs) from healthy donors (HDs) to provide the preliminary and descriptive evidence that AIRE could bind to the promoters of known autoantigens in human peripheral blood.

## 2. Results

### 2.1. AIRE Binds to Promoter Regions of Thyroid and Pancreatic Autoantigens in Human PBMCs

**Figure 1:**
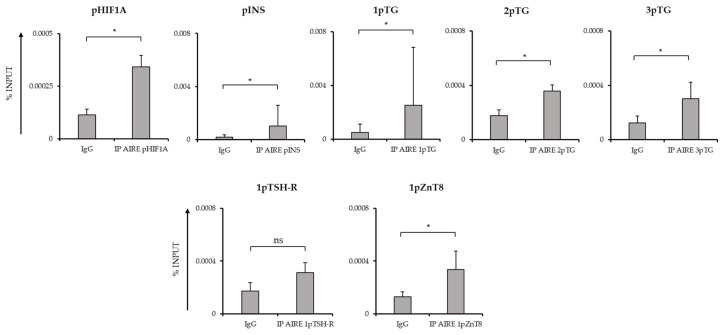
Cross-linked protein DNA complexes from HD69, HD72, HD73, and HD74 PBMC were subjected to chromatin immunoprecipitation (ChIP) using an anti- autoimmune regulator (AIRE) antibody, and analyzed using quantitative real-time PCR (qRT-PCR). Immunoprecipitations with corresponding IgG isotype controls were performed in parallel. ChIP results are shown as % input DNA. Data represent mean values ± SD of at least three biological replicates. Statistical significance was assessed by paired Student’s *t*-test. * *p* < 0.05.

ChIP of PBMC samples from four healthy control individuals with anti-AIRE antibodies showed amplification of promoters of thyroglobulin (*TG*), thyrotropin (*TSH*) receptor (*TSH-R*), insulin (*INS*) and zinc transporter 8 (*ZnT8*), compared to negative control IgG (immunoglobulin G). The promoter regions of the selected autoantigens were sequenced and validated using specific primers (see Section 4) before the ChIP experiments were carried out. Input chromatin was quantified, and the quality of the isolated chromatin was consistently reproducible across experiments. Washing and purification steps were accurately performed. ChIP results for the four samples, expressed as percent input (% input) values, revealed amplicons of *INS*, *TG*, *TSH-R* and *ZnT8*, thus identifying these autoantigen promoters as direct targets of AIRE in PBMC samples from healthy controls (Figure 1). We observed significant AIRE enrichment at the promoter regions of the autoantigens *INS*, *ZnT8* and at three different promoter regions of *TG* (1p, 2p and 3p) compared to IgG controls (Figure 1). Furthermore, quantification of ChIP signals was also estimated as ng of DNA recovered after AIRE immunoprecipitation, as illustrated in Supplementary Figure S1.

**Figure 2:**
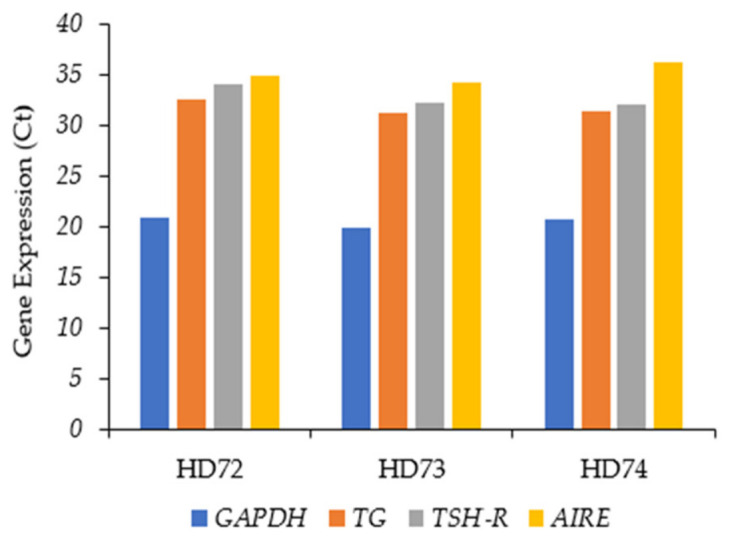
qRT-PCR analysis. Gene expression of *AIRE*, *TG* and *TSH-R*, compared to *GAPDH*, in representative samples HD72, HD73 and HD74.

Quantitative real-time PCR (qRT-PCR) on PBMCs from samples HD72, HD73, and HD74 confirmed *AIRE* expression and the most abundant corresponding transcripts of the tested representative *TG* and *TSH-R* autoantigens (Figure 2).

### 2.2. AIRE Expression Detected in Human Bulk PBMC Samples Using Western Blot Analysis

**Figure 3:**
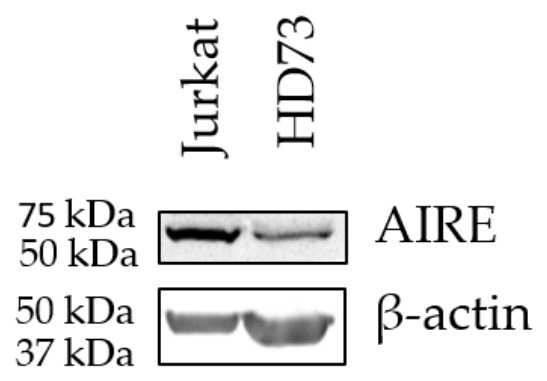
Protein expression level of AIRE in HD73 PBMC sample in comparison with Jurkat T-cell control using the anti-AIRE monoclonal antibody (MoAb) (Cat. sc-373703, clone H-300, mouse IgG_1_κ, Santa Cruz Biotechnology, Dallas, TX, USA). The MoAb depicts a band of the expected size of approximately 55 KDa. β-actin is used as loading control.

Western blot analysis confirmed that AIRE was expressed at the protein level in the residual HD73 PBMC sample (Figure 3). Moreover, AIRE expression was consistently detected in an additional six bulk PBMC samples from HDs (Supplementary Figure S2).

## 3. Discussion

Potential mechanisms underlying the AIRE’s contribution to peripheral tolerance have been reported in the literature, because its expression correlates with a more mature DC phenotype [8]. As regards the level of AIRE expression, this can vary substantially in DCs, from transient expression to a temporal state of maturation. Evidence for a more direct role of AIRE in DC maturation derives from studies on AIRE-deficient cells from APECED patients, where monocyte-derived DCs (moDCs) exhibited impaired function as well as impaired maturation [14,15,16]. Conversely, AIRE is not crucial for the development of tolerogenic DCs, as these can be generated in vitro from APECED monocytes, and their role and function in Type 1 regulatory T-cell (Tr1) differentiation are similar to their role and function in tolerogenic DCs generated from HDs [17]. Furthermore, there are clues suggesting that AIRE might contribute to peripheral tolerance through the exhaustion of effector T-cells. Indeed, in non-obese diabetic mice (NOD) [18], DC-specific overexpression of AIRE results in a decrease in interferon gamma (IFN-γ) and tumor necrosis factor alpha (TNF-α) secretion by T-cells. This is accompanied by increased expression of exhaustion-associated markers such as PD1 (programmed death 1), LAG3 (lymphocyte activation gene 3) and TIM3 (T-cell immunoglobulin and mucin domain 3) without significant changes in the population of FoxP3^+^ Tregs. Indeed, even in mice, certain lymph node stromal cells residing in the lymph node cortex can express *Aire*. Strikingly, there is evidence that these cells also express specific tissue-restricted antigens (TRAs), including antigens from the eye, intestine, pancreas, liver, central nervous system and skin that could potentially be used for presentation and tolerance [19].

An important point to note, as reported in Section 1 (*vide supra*), is that emerging evidence from human studies does indeed support a role for extrathymic AIRE-expressing cells in peripheral tolerance. AIRE-expressing dendritic cells have been identified in human peripheral lymphoid tissues, particularly in areas associated with antigen entry and with initiation of immune responses. These cells express tissue-specific antigens together with tolerogenic molecules such as IDO and IL-10, suggesting that AIRE might contribute to peripheral tolerance in humans [20]. Moreover, gene expression analysis of human fetal liver has detected AIRE and tissue-specific antigen gene expression in CD71^+^ erythroid cells [21]. eTACs might contribute to the maintenance of self-tolerance in humans through mechanisms such as Treg induction or deletion of autoreactive T-cells [21].

In the present work, the issue of AIRE binding to promoters of known autoantigens was explored through ChIP, a valuable tool in studying gene regulation, including chromatin remodeling, histone modifications and protein/DNA interactions, methodologies that are also applied in the field of immunology [22]. An anti-human AIRE monoclonal antibody (MoAb) was used to bind and immunoprecipitate high-purity chromatin which was extracted after formaldehyde cross-linking to enable preservation. DNA isolated through reverse crosslinking and precipitated material was then analyzed using qRT-PCR [23]. Standardization of this approach is remarkably challenging, especially those steps involving quantitative analysis of the precipitate and normalization of data. Extraction of good chromatin relies heavily on the quality of the starting material, generally obtained from human PBMCs that are rescued using standardized methods. Shearing of chromatin was then achieved through sonication (hydrodynamic shearing) followed by estimation of sonication efficiency using gel electrophoresis [23]. For ChIP, we relied on the validated experience of other groups using bulk PBMC samples as starting material, where low AIRE expression [11] is detected when employing the same anti-AIRE MoAb. Indeed, monoclonal antibodies are at higher specificity compared to polyclonal sera which can recognize several epitopes of the target, increasing the chance that low-abundance templates might be selected [24]. With this MoAb, we would expect to address the fact that different antibodies can differ in their sensitivity to inhibitory factors present in the input chromatin samples. Appropriate controls were introduced by analyzing in parallel the input sample and the control sample to which the normal goat IgG was added, in order to estimate the amount of background signal relative to the ‘true signal’ in the subsequent qRT-PCR.

Normalization of obtained data precedes data interpretation. Therefore, choosing an appropriate normalization method is essential, because this can influence the final conclusions. As shown in Figure 1, qRT-PCR results are presented as % input, calculated by comparing the number of target genomic regions detected in the AIRE immunoprecipitation to the corresponding input. IgG immunoprecipitation was included as a negative control. ChIP analysis demonstrated the physical occupancy of AIRE at specific autoantigen promoter regions, and enrichment at the loci of interest was assessed relative to a negative control locus (genomic desert region). This result of enrichment was confirmed by statistical analysis. This approach demonstrates that the observed signal is specific to the target regions, exceeds background, and is reproducible across all four healthy donor samples.

It is important to note that qRT-PCR experiments confirmed not only that there was *AIRE* expression in PBMC from available samples, but also expression of some representative autoantigens (including *TG* and *TSH-R*) among those evaluated in this investigation. Thus, the transcriptional results support the biological relevance of the identified AIRE-bound loci. Furthermore, AIRE expression was detected at the protein level in the only residual PBMC sample (HD73) of the four original samples, reinforcing the identification of AIRE-bound regions in autoantigen promoter genes. This result was confirmed across all additional bulk PBMC samples from the healthy donors that could be recruited. These findings provide evidence that AIRE is expressed at both the transcriptional and protein level in PBMC. Together, these results strengthen the evidence that the occupancy of AIRE at the autoantigens, detected by ChIP-qPCR, is physiologically relevant, and not the result of a nonspecific chromatin interaction. Although these findings are consistent with a potential role of AIRE in the regulation of the investigated autoantigen genes, further functional studies will be necessary to determine whether *AIRE* is transcriptionally and immunologically active in human peripheral blood, and to clarify the AIRE underlying molecular and immunological mechanisms.

Considering the high number of cells (approximately 30 × 10^6^) necessary to carry out this experiment through chromatin immunoprecipitation with the established protocol using a validated anti-AIRE MoAb, it would not be feasible to work on sorted single AIRE-positive subsets. Therefore, the AIRE ChIP signal detected in PBMC most likely originates from a small cellular subpopulation within the heterogeneous PBMC compartment, such as eTACs, DCs, or B-cells. However, the correlation of AIRE expression and tissue restricted autoantigens in sorted AIRE-positive subsets determined by qRT-PCR has already been reported in published studies (*vide supra*) [20,21]. Furthermore, in this preliminary investigation, we were able to rely on a small number of samples (n = 4) from normal donors for which the recovered quantity of cells was valid for the purposes of carrying out the experiment. In conclusion, although AIRE-mediated regulation of tissue-restricted autoantigens has been extensively characterized in thymic epithelial cells, evidence of AIRE occupancy at the autoantigen promoter regions in human peripheral blood cells remains to be fully elucidated. Therefore, the present study reports the preliminary and descriptive evidence for AIRE occupancy at promoter regions of known autoantigens in human bulk PBMC from healthy donors. On a general ground, the phenomenon of AIRE occupancy at promoter regions may vary in extent and specificity across different autoantigens and among donors, highlighting the need for further validation in larger cohorts. These results of the present study open the pathway to future investigations aiming to describe subset-level expression of selected autoantigens in sorted AIRE-positive versus AIRE-negative PBMC populations.

## 4. Materials and Methods

### 4.1. Human PBMC Recruitment and Sample Collection

To unravel the *AIRE* mechanism in the pathogenesis of pediatric autoimmune diseases, due to the limited amount of blood that can be obtained from children, we had to perform the study using samples from adult human donors, these being available in larger quantity.

PBMCs were separated by Ficoll-Hypaque (Histopaque, Sigma-Aldrich Chemical C, St. Louis, MO, USA) from 50 mL sodium-heparinized venous buffy coat samples taken from four HDs, washed twice in phosphate-buffered saline (PBS) (Lonza, Verviers, Belgium), and then frozen in liquid nitrogen.

HD subjects (n = 4) were enrolled in the investigation after being recruited at the OPBG Blood Transfusion Centre. The study was approved by the OPBG Ethics Committee under Study number 1275_opbg_2016 (approved on 20 March 2017). Written informed consent from human donors was obtained.

### 4.2. Chromatin Immunoprecipitation

ChIP of PBMC was performed using an Active Motif ChIP-IT^®^ PBMC Kit (Vinci Biochem Srl, Florence, Italy) according to the protocol indicated by the manufacturer. Briefly, approximately 30 × 10^6^ PBMC were fixed in formaldehyde (Sigma-Aldrich, St. Louis, MO, USA) for protein–DNA cross-linking at room temperature (RT) for 15 min (min), and the reaction was stopped by adding the solution provided by the kit. Cells were then lysed and sheared to obtain chromatin fragments by sonicating in a Bioruptor Plus Diagenode (Liege, Belgium) in a 4 °C water bath at high power for 10 cycles of 10 s, repeated for three rounds, with vortexing and brief centrifugation between rounds to ensure homogeneous fragmentation. Samples were subsequently centrifuged at 18,000× *g* for 5 min at 4 °C to remove cellular debris. The size and quality of chromatin fragmentation were verified on 2% agarose gels (Supplementary Figure S3). Chromatin was quantified using a Qubit dsDNA BR assay (Sigma-Aldrich). For each immunoprecipitation reaction, 30 μg of chromatin diluted in 650 μL of ChIP buffer (provided in the Active Motif ChIP-IT^®^ PBMC Kit) was incubated with Protein G-coated magnetic beads and 7 μg of anti-human AIRE monoclonal (mouse) antibody (Cat. sc-373703, RRID: not available (N/A), Santa Cruz Biotechnology, Santa Cruz, CA, USA) at 4 °C overnight (ON) in an end-to-end rotator. For negative control, 8 μg of IgG1 goat serum (Sigma-Aldrich) per 30 μg of chromatin was employed. 50 μg/μL of sheared chromatin was used for the input fraction. As control for the performance of ChIP technology for active chromatin immunoprecipitation, we used anti-human H3 rabbit polyclonal antibodies (acetyl K27) (Cat. ab4729, RRID: AB_2118291, Abcam, Cambridge, UK) (2 μg per 25 μg of chromatin). Genomic DNA from beads was eluted and purified, and subsequently analyzed by qRT-PCR. Enrichment of the hypoxia-inducible factor 1 alpha (*HIF1A*) promoter was assessed as a positive control region of immunoprecipitation, known to have a non-canonical AIRE binding site as a direct target of AIRE [24]. A gene desert region known to be devoid of gene-encoding proteins was used as negative control. qRT-PCR was then performed to evaluate AIRE binding at the promoter regions of selected autoantigen genes, including *TG*, *TSH-R* [25], and insulin-dependent diabetes (Type 1 diabetes)-related autoantigens, namely, *INS* and *ZnT8* [26] (Supplementary Table S1). Primer efficiencies were assumed to be close to 100%, and the promoter regions of the selected autoantigens were previously sequenced and validated using these primers, thus confirming their specificity (*vide supra*). PCR reactions were run at 94 °C × 2 min, followed by 40 cycles at 94 °C for 30 s, three different annealing temperatures (55 °C, 57 °C and 59 °C) for 30 s and 72 °C for 30 s, followed by extension at 72 °C for 5 min [27]. Four independent experiments were conducted to validate the result. Recovered nanograms of DNA from immunoprecipitation were estimated by Nanodrop and Qubit dsDNA BR Assay kit (Invitrogen, Carlsbad, CA, USA). Only those ChIP experiments in which the immunoprecipitated DNA showed an enrichment of ≥2% input at the *HIF1A* promoter region were considered successful. qRT-PCR was performed in three technical replicates for each sample, and Ct values were averaged prior to analysis. Melting curve analysis was performed for each primer pair and confirmed the amplification of a single, specific product. qRT-PCR values were presented as % input, calculated by comparing the number of target genomic regions detected in the immunoprecipitated samples relative to the corresponding input sample [28]. Measurements were taken in four independent healthy donors.

### 4.3. Real-Time PCR

RNA was extracted from PBMC samples from the same healthy blood donors used for ChIP using RNeasy Plus Mini Kit (QIAgen, Milan, Italy) according to the manufacturer’s guidelines. RNA was quantified using a Nanodrop spectrophotometer, and reverse transcribed using the Superscript IV First Strand Synthesis Kit (Thermo Fisher Scientific Srl, Milan, Italy). The following representative gene transcripts were analyzed on RNA from residual PBMC samples: thyroglobulin using forward CCAGTGGCTTCTCTTCCTGACT and reverse CCTTGGAGGAAGCGGATGGTTT primers; and TSH-R using forward GAGTTTCCTTCACCTCACACGG and reverse CTGCTCTCATTACACATCAAGGAC primers. Measurements were taken in technical triplicates, and data reported in figures as mean Ct values.

### 4.4. Protein Extraction and Western Blot Analysis

PBMC and Jurkat cells were lysed in RIPA Lysis Buffer 10x (Merck, Darmstadt, Germany) and incubated on ice for 15 min. The samples were then centrifuged at 18,000× *g* for 15 min at 4 °C to remove cellular debris, and the supernatants were collected in new tubes. Protein concentrations of cell lysates were determined by spectrophotometric analysis using the Bradford dye reagent (Sigma-Aldrich). Before immunoblotting, samples were supplemented with β-mercaptoethanol (Sigma-Aldrich) and boiled for 10 min at 100 °C. Protein extracts were run on SDS-polyacrylamide gels and then transferred to nitrocellulose membranes (Biorad, Hercules, CA, USA). Membranes were blocked at RT for 1 h with 5% bovine serum albumin (Sigma-Aldrich) in Tris-buffer saline (TBS) (Biorad) containing 0.1% Tween-20 (Sigma-Aldrich). Next, membranes were incubated at 4 °C ON with primary antibodies against β-actin (Cat. AC050, RRID: N/A, Aurogene S.r.l., Rome, Italy) and AIRE (Santa Cruz Biotechnology), followed by incubation with HRP-conjugated secondary antibodies: anti-rabbit (Biorad, Hercules, CA, USA) or anti-mouse (Biorad). Immunoreactive bands were detected by enhanced chemiluminescence using the ECL Western Blotting Analysis System (Cytiva, Marlborough, MA, USA) and visualized using the iBright 1500 Imaging System (Invitrogen, Carlsbad, CA, USA).

### 4.5. Statistical Analysis

Statistical tests were performed by using GraphPad Prism software version 10 (GraphPad Software, San Diego, CA, USA). Statistical data analysis between two groups was carried out using a two-tailed Student’s paired *t*-test. Differences were considered statistically significant when *p*-values were <0.05.

## Data Availability

The datasets presented in this article are not readily available because of requirements of ethical consent for healthy human donors. Requests to access the datasets should be directed to the corresponding author.

## Supplementary Materials

The following supporting information can be downloaded at: https://www.mdpi.com/article/10.3390/ijms27135807/s1.

## Abbreviations

The following abbreviations are used in this manuscript:

TCR	T-cell receptor
*AIRE*	AutoImmune REgulator gene
mTECs	medullary Thymic Epithelial Cells
APECED	Autoimmune PolyEndocrinopathy-Candidiasis-Ectodermal Dystrophy
APS1	Autoimmune Polyendocrine Syndrome Type 1
APC	Antigen-Presenting Cell
DCs	Peripheral tolerogenic Dendritic Cells
eTACs	extraThymic AIRE-expressing Cells
IDO	Indoleamine 2,3 dioxygenase
IL-10	Interleukin 10
PD-L1	Programmed Death-Ligand 1
MH class II	Major Histocompatibility Complex Class II
Tregs	T regulatory cells
ChIP	Chromatin Immunoprecipitation
PBMCs	Peripheral Blood Mononuclear Cells
HDs	Healthy Donors
N/A	Not available
*TG*	Thyroglobulin
*TSH*	Thyrotropin
*TSH-R*	Thyrotropin Receptor
*INS*	Insulin
*ZnT8*	Zinc Transporter 8
IgG	Immunoglobulin G
AT	Annealing Temperature
% input	Percent Input
Des	Desert
qRT-PCR	Quantitative Real-Time PCR
moDCs	Monocyte-derived Dendritic Cells
Tr1	Type 1 regulatory T-cell
NOD	Non-Obese Diabetic mice
INF-γ	Interferon gamma
TFN-α	Tumor Necrosis Factor alpha
PD1	Programmed Death 1
LAG3	Lymphocyte Activation Gene 3
TIM3	T-cell Immunoglobulin and Mucin domain 3
MoAb	Monoclonal Antibody
TRA	Tissue-Restricted Antigens
PBS	Phosphate Buffer Saline
RT	Room Temperature
Min	Minutes
ON	Overnight
HIF1A	Hypoxia-Inducing Factor 1A
pTG	Thyroglobulin Promoter
pTSH-R	Thyrotropin Receptor Promoter
pINS	Insulin Promoter
pZnT8	Zinc Transporter 8 Promoter
TBS	Tris-buffer saline

## References

[1] Rosenblum M.D., Remedios K.A., Abbas A.K. (2015). Mechanisms of human autoimmunity. J. Clin. Investig..

[2] Metzger T.C., Anderson M.S. (2011). Control of central and peripheral tolerance by Aire. Immunol. Rev..

[3] Washburn T., Schweighoffer E., Gridley T., Chang D., Fowlkes B.J., Cado D., Robey E. (1997). Notch activity influences the alphabeta versus gammadelta T cell lineage decision. Cell.

[4] Fierabracci A. (2011). Recent insights into the role and molecular mechanisms of the autoimmune regulator (AIRE) gene in autoimmunity. Autoimmun. Rev..

[5] Perniola R. (2018). Twenty years of AIRE. Front. Immunol..

[6] Gardner J.M., DeVoss J.J., Friedman R.S., Wong D.J., Tan Y.X., Zhou X., Johannes K.P., Su M.A., Chang H.Y., Krummel M.F. (2008). Deletional tolerance mediated by extrathymic Aire-expressing cells. Science.

[7] Hamilton-Williams E.E., Bergot A.S., Reeves P.L., Steptoe R.J. (2016). Maintenance of peripheral tolerance to islet antigens. J. Autoimmun..

[8] van Laar G.G., van Hamburg J.P., Tas S.W. (2022). Extrathymic AIRE-expressing cells: Friends or foes in autoimmunity and cancer?. Autoimmun. Rev..

[9] Tas S.W., Vervoordeldonk M.J., Hajji N., Schuitemaker J.H.N., van der Sluijs K.F., May M.M., Ghosh S., Kapsenberg M.L., Tak P.P., de Jong E.C. (2007). Noncanonical NF-kappaB signaling in dendritic cells is required for indoleamine 2,3-dioxygenase induction and immune regulation. Blood.

[10] Gardner J.M., Metzger T.C., McMahon E.J., Au-Yeung B.B., Krawisz A.K., Lu W., Price J.D., Johannes K.P., Satpathy A.T., Murphy K.M. (2013). Extrathymic Aire-expressing cells are a distinct bone marrow-derived population that induce functional inactivation of CD4^+^ T cells. Immunity.

[11] Suzuki E., Kobayashi Y., Kawano O., Endo K., Haneda H., Yukiue H., Sasaki H., Yano M., Maeda M., Fujii Y. (2008). Expression of AIRE in thymocytes and peripheral lymphocytes. Autoimmunity.

[12] Bergström B., Lundqvist C., Vasileiadis G.K., Carlsten H., Ekwall O., Ekwall A.H. (2019). The rheumatoid arthritis risk gene AIRE is induced by cytokines in fibroblast-like synoviocytes and augments the pro-inflammatory response. Front. Immunol..

[13] Husebye E.S., Anderson M.S., Kämpe O. (2018). Autoimmune polyendocrine syndromes. N. Engl. J. Med..

[14] Pöntynen N., Strengell M., Sillanpää N., Saharinen J., Ulmanen I., Julkunen I., Peltonen L. (2008). Critical immunological pathways are downregulated in APECED patient dendritic cells. J. Mol. Med..

[15] Sillanpää N., Magureanu C.G., Murumägi A., Reinikainen A., West A., Manninen A., Lahti M., Ranki A., Saksela K., Krohn K. (2004). Autoimmune regulator induced changes in the gene expression profile of human monocyte-dendritic cell lineage. Mol. Immunol..

[16] Ryan K.R., Hong M., Arkwright P.D., Gennery A.R., Costigan C., Dominguez M., Denning D., McConnell V., Cant A.J., Abinun M. (2008). Impaired dendritic cell maturation and cytokine production in chronic mucocutaneous candidiasis with or without APECED. Clin. Exp. Immunol..

[17] Crossland K.L., Abinun M., Arkwright P.D., Cheetham T.D., Pearce S.H., Hilkens C.M.U., Lilic D. (2016). AIRE is not essential for the induction of human tolerogenic dendritic cells. Autoimmunity.

[18] Kulshrestha D., Yeh L.T., Chien M.W., Chou F.C., Sytwu H.K. (2017). Peripheral autoimmune regulator induces exhaustion of CD4^+^ and CD8^+^ effector T cells to attenuate autoimmune diabetes in non-obese diabetic mice. Front. Immunol..

[19] Ovcharenko I., Loots G.G., Nobrega M.A., Hardison R.C., Miller W., Stubbs L. (2005). Evolution and functional classification of vertebrate gene deserts. Genome Res..

[20] Poliani P.L., Kisand K., Marrella V., Ravanini M., Notarangelo L.D., Villa A., Facchetti F. (2010). Human peripheral lymphoid tissues contain autoimmune regulator-expressing dendritic cells. Am. J. Pathol..

[21] Perik-Zavodskii R., Perik-Zavodskaya O., Shevchenko Y., Alrhmoun S., Volynets M., Zaitsev K., Sennikov S. (2022). Human fetal liver parenchyma CD71^+^ cells have AIRE and tissue-specific antigen gene expression. Genes.

[22] Carey M.F., Peterson C.L., Smale S.T. (2009). Chromatin immunoprecipitation (ChIP). Cold Spring Harb. Protoc..

[23] Haring M., Offermann S., Danker T., Horst I., Peterhansel C., Stam M. (2007). Chromatin immunoprecipitation: Optimization, quantitative analysis and data normalization. Plant Methods.

[24] Lee J.W., Epardaud M., Sun J., Becker J.E., Cheng A.C., Yonekura A.R., Heath J.K., Turley S.J. (2007). Peripheral antigen display by lymph node stroma promotes T cell tolerance to intestinal self. Nat. Immunol..

[25] McLachlan S.M., Rapoport B. (2014). Breaking tolerance to thyroid antigens: Changing concepts in thyroid autoimmunity. Endocr. Rev..

[26] Arvan P., Pietropaolo M., Ostrov D., Rhodes C.J. (2012). Islet autoantigens: Structure, function, localization, and regulation. Cold Spring Harb. Perspect. Med..

[27] Padmanabhan R.A., Johnson B.S., Dhyani A.K., Pillai S.M., Jayakrishnan K., Laloraya M. (2023). Autoimmune regulator (AIRE) takes a hypoxia-inducing factor 1A route to regulate FOXP3 expression in PCOS. Am. J. Reprod. Immunol..

[28] Solomon E.R., Caldwell K.K., Allan A.M. (2021). A novel method for the normalization of ChIP-qPCR data. MethodsX.

