# Supplementary material for: Preliminary Evidence for Autoimmune Regulator Occupancy at Promoter Regions of Known Autoantigens in Human Peripheral Lymphocytes Obtained by Chromatin Immunoprecipitation Assay
Source: Int J Mol Sci. 2026 Jun 26;27(13):5807. doi: 10.3390/ijms27135807 (PMC13360696; doi:10.3390/ijms27135807)
Supplement: Supplementary file 1 [file ijms-27-05807-s001.zip › Supplementary Figure S1.pdf]

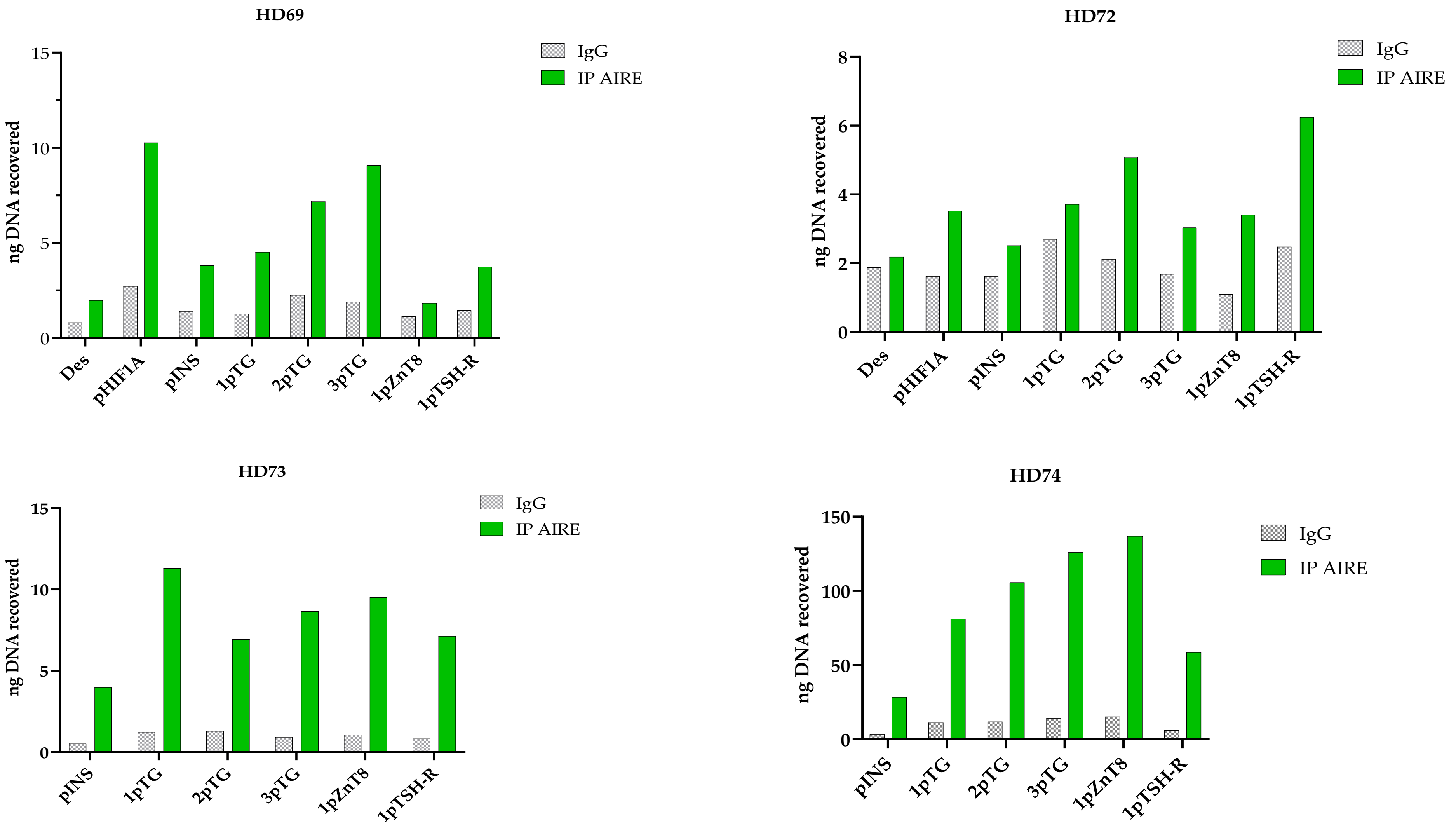

Supplementary Figure S1. ChIP-qRT-PCR analysis results of samples HD69, HD72, HD73 and HD74. Levels of INS, TG, ZnT8 and TSH-R autoantigens after AIRE and corresponding IgG isotype control immunoprecipitation are expressed as recovered nanograms (ng) of DNA.
