# Supplementary material for: Preliminary Evidence for Autoimmune Regulator Occupancy at Promoter Regions of Known Autoantigens in Human Peripheral Lymphocytes Obtained by Chromatin Immunoprecipitation Assay
Source: Int J Mol Sci. 2026 Jun 26;27(13):5807. doi: 10.3390/ijms27135807 (PMC13360696; doi:10.3390/ijms27135807)
Supplement: Supplementary file 1 [file ijms-27-05807-s001.zip › supplementary Figure S2.pdf]

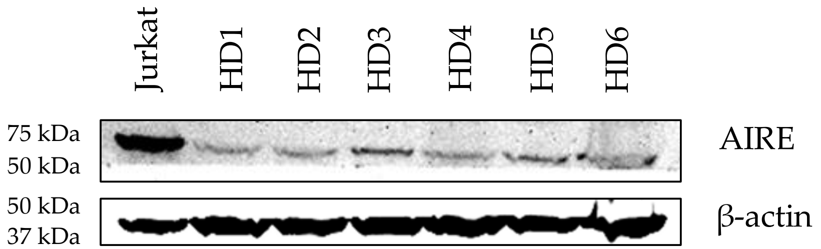

Supplementary Figure S2. Protein expression levels of AIRE in representative HD1, HD2, HD3, HD4, HD5 and HD6 samples in comparison with Jurkat T-cell. β-actin is used as loading control.
