# Supplementary material for: Preliminary Evidence for Autoimmune Regulator Occupancy at Promoter Regions of Known Autoantigens in Human Peripheral Lymphocytes Obtained by Chromatin Immunoprecipitation Assay
Source: Int J Mol Sci. 2026 Jun 26;27(13):5807. doi: 10.3390/ijms27135807 (PMC13360696; doi:10.3390/ijms27135807)
Supplement: Supplementary file 1 [file ijms-27-05807-s001.zip › Supplementary Figure S3.pdf]

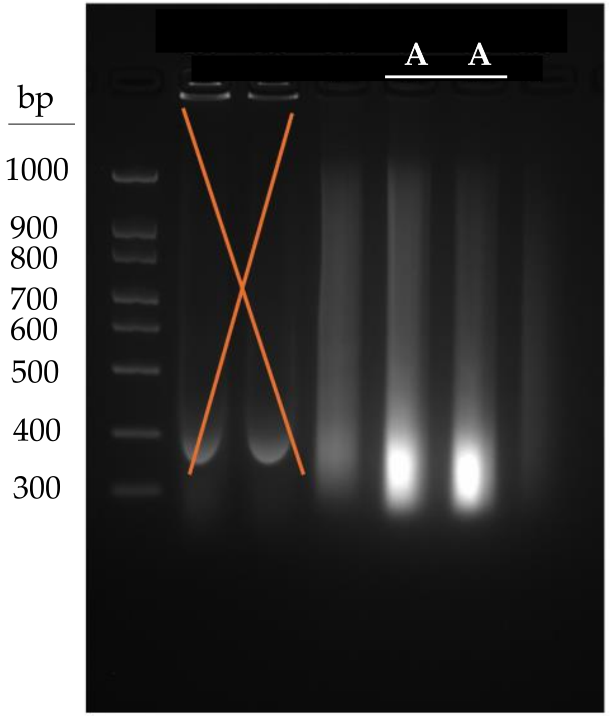

Supplementary Figure S3. DNA fragmentation profile of a representative ChIP sample (A) run in technical duplicate showing fragment sizes in the length range of 300-400bp, analyzed by electrophoresis on a 2% agarose gel.
