# Supplementary material for: Preliminary Evidence for Autoimmune Regulator Occupancy at Promoter Regions of Known Autoantigens in Human Peripheral Lymphocytes Obtained by Chromatin Immunoprecipitation Assay
Source: Int J Mol Sci. 2026 Jun 26;27(13):5807. doi: 10.3390/ijms27135807 (PMC13360696; doi:10.3390/ijms27135807)
Supplement: Supplementary file 1 [file ijms-27-05807-s001.zip › Supplementary Table S1.pdf]

Gene Name	Primer Name	Sequences 5′- 3′	Annealing Temperature
HIF1A	*HIF1A*-Forward	CCCTTGAAGTTTACAGCAACAG	57°C
	HIF1A-Reverse	ACAGGGGAACTCACCTTGTC	
Desert	*Desert*-Forward	CCCAAACTCTGAGAGGCTTATT	58°C
	Desert-Reverse	GAGCCATCATCTAGACACCTTC	
1pTG	*1pTG*-Forward	CAGGTCTCACAGGAACAG	50°C
	1pTG-Reverse	CAACCAAAAGATAGCAGG	
2pTG	*2pTG*-Forward	CCTGCTATCTTTTGGTTG	51.5°C
	2pTG-Reverse	GGTGAGGGAAGCAAAATAC	
3pTG	*3pTG*-Forward	GAAGGAGAAGGAGAAAGGGTAG	55°C
	3pTG-Reverse	GTATCTTTGGAGGGAACAGG	
1pTSH-R	*1pTSH-R*-Forward	CCATTATCTAGTCGCGAG	50°C
	1pTSH-R -Reverse	CTGGAGGTTAAGTGGATG	
pINS	*pINS*-Forward	GAGACATTTGCCCCCAGC	60°C
	pINS-Reverse	CGTCAGCACCTCTTCCTCAG	
1pZnT8	*1pZnT8*-Forward	CTAGTTATCCTTGTGGTCAC	50°C
	1pZnT8-Reverse	CATAGGGTTATTGGGAG	

Supplementary Table S1. Sequences and annealing temperatures of primers used for qRT-PCR.
